# Perturbation Analysis of a Prognostic *DDX3X*-Mediated Gene Expression Signature Identifies the Antimetastatic Potential of Chaetocin in Hepatocellular Carcinoma

**DOI:** 10.3390/cells12121628

**Published:** 2023-06-14

**Authors:** Tsung-Chieh Lin

**Affiliations:** 1 Genomic Medicine Core Laboratory, Department of Medical Research and Development, Chang Gung Memorial Hospital, Linkou, Taoyuan City 333, Taiwan; tclin1980@cgmh.org.tw; Tel.: +886-3-3281200 (ext. 7722); 2 Department of Biomedical Sciences, Chang Gung University, Taoyuan City 333, Taiwan

**Keywords:** *DDX3X*, chaetocin, hepatocellular carcinoma

## Abstract

ATP-dependent RNA helicase DDX3X, also known as DEAD (Asp-Glu-Ala-Asp) Box Polypeptide 3, X-Linked (DDX3X), is critical for RNA metabolism, and emerging evidence implicates ATP-dependent RNA helicase DDX3X’s participation in various cellular processes to modulate cancer progression. In this study, the clinical significance of *DDX3X* was addressed, and *DDX3X* was identified as a biomarker for poor prognosis. An exploration of transcriptomic data from 373 liver cancer patients from The Cancer Genome Atlas (TCGA) using Ingenuity Pathway Analysis (IPA) suggested an association between *DDX3X* expression and cancer metastasis. Lentiviral-based silencing of *DDX3X* in a hepatocellular carcinoma (HCC) cell line resulted in the suppression of cell migration and invasion. The molecular mechanism regarding ATP-dependent RNA helicase DDX3X in liver cancer progression had been addressed in many studies. I focused on the biological application of the *DDX3X*-mediated gene expression signature in cancer therapeutics. An investigation of the *DDX3X*-correlated expression signature via the L1000 platform of Connectivity Map (BROAD Institute) first identified a histone methyltransferase inhibitor, chaetocin, as a novel compound for alleviating metastasis in HCC. In this study, the prognostic value of *DDX3X* and the antimetastatic property of chaetocin are presented to shed light on the development of anti-liver cancer strategies.

## 1. Introduction

Emerging evidence indicates the critical regulatory role of DEAD (Asp-Glu-Ala-Asp) Box Polypeptide 3, X-Linked (DDX3X) in cancer progression. ATP-dependent RNA helicase DDX3X, a RNA helicase, is a DEAD-box family member, and has been reported to be involved in the splicing of pre-mRNA [1], RNA export [2], the transcription of RNA [3], and protein translation [4,5,6]. Due to its complex biological role in RNA metabolism, ATP-dependent RNA helicase DDX3X has gained increasing attention for its function in many types of cancer, and it regulates tumor progression in a complex manner. Furthermore, this complexity is further increased because ATP-dependent RNA helicase DDX3X generally exhibits its biological effects as components of multiprotein complexes [7]. The exact impacts of ATP-dependent RNA helicase DDX3X are affected by its interacting partners [8]. The overexpression of ATP-dependent RNA helicase DDX3X has been detected in hepatocellular carcinoma (HCC), and ATP-dependent RNA helicase DDX3X has been characterized as a critical gene in hepatocarcinogenesis [9]. In contrast, the loss of ATP-dependent RNA helicase DDX3X was found to lead to tumorigenesis due to a reduction in the expression of DNA repair factors in a mouse model [10]. Other studies revealed the loss of *DDX3X* expression in hepatocellular carcinoma tissue, and increased tumor cell proliferation because of the *DDX3X* silencing in hepatocellular carcinomas which were infected by hepatitis virus [3,11]. Hence, the conflicting roles of ATP-dependent RNA helicase DDX3X are inconsistent within the same type of cancer.

These conflicting results emphasize the urgent need for clarification. The prognostic role of *DDX3X* is a critical factor in determining whether *DDX3X* plays an oncogenic or tumor-suppressive role. In this study, I first aimed to characterize *DDX3X*’s role from the perspective of its prognostic significance in hepatoma patient survival. The current updated version of the liver cancer/hepatoma cohort, with more patients enrolled, was retrieved from The Cancer Genome Atlas (TCGA) database and reanalyzed. This analysis of the *DDX3X*-associated gene signature further suggested the potential value of studying its biological function in promoting hepatoma metastasis. Furthermore, ATP-dependent RNA helicase DDX3X has been proposed as a therapeutic target for cancers [12]. I also aimed to identify a novel use for a drug in reversing the *DDX3X*-mediated gene signature via a Connectivity Map (BROAD Institute) [13]. In this study, I demonstrated the prognostic value of *DDX3X* and the potential capability of a histone methyltransferase inhibitor, chaetocin, as a therapeutic target to shed light on the development of anti-liver cancer strategies.

## 2. Materials and Methods

### 2.1. TCGA Dataset Analysis

Gene expression in the TCGA liver hepatocellular carcinoma (LIHC) dataset (Dataset ID: TCGA.LIHC.sampleMap/HiSeqV2_PANCAN, version: 2017-10-13) was estimated by RNA-Seq (Illumina HiSeq), and these data were retrieved along with the associated clinical information for data analysis. The RNA-Seq read count was normalized and log2-transformed. A total of 373 HCC cases were divided into a *DDX3X*-high group and a *DDX3X*-low group based on the ranking assigned by the *DDX3X* expression level.

### 2.2. Ingenuity Pathway Analysis

The differential gene expression signatures in the HCC cohort were obtained after dividing the cohort into two groups with relatively high and low DDX3X levels. The gene signatures were further analyzed using Ingenuity^®^ Pathway Analysis (IPA; QIAGEN, Hilden, Germany; https://digitalinsights.qiagen.com/products-overview/discovery-insights-portfolio/analysisand-visualization/qiagen-ipa/ (accessed on 1 November 2022)) according to the instructions provided. A list of relevant networks, upstream regulators and algorithmically generated mechanistic networks based on connectivity was obtained upon the comparison of the Ingenuity^®^ Knowledge Database with the imported dataset. The canonical pathway analysis feature of IPA was also used to identify significant diseases and functions in rank order based on the altered gene signatures.

### 2.3. Cell Culture

Human hepatocellular carcinoma cell lines were purchased from Bioresource Collection and Research Center (Hsinchu, Taiwan). HA 22T/VGH and HA 59T/VGH cells were maintained in DMEM supplemented with 10% FBS, 0.1 mM NEAA, penicillin (100 units/mL), and streptomycin (100 µg/mL). C3A (HepG2/C3A) and HepG2 cells were maintained in MEM supplemented with 10% FBS, 0.1 mM NEAA, 1 mM sodium pyruvate, penicillin (100 units/mL), and streptomycin (100 µg/mL). The cells were incubated in a humidified atmosphere of 95% air and 5% CO_2_ at 37 °C. Chaetocin was purchased from Santa Cruz (CAS 28097-03-2, Dallas, TX, USA). The compound was dissolved in DMSO. In the chaetocin pretreatment assay, cancer cells were incubated with indicated concentrations of chaetocin for 48 h, and cells were then subjected to migration/invasion/wound-healing assay after removing chaetocin.

### 2.4. Lentivirus-Based shRNA Production and Infection

The lentiviral shRNA constructs were purchased from Thermo Scientific (Pittsburgh, PA, USA). Lentiviruses were produced via co-transfection of 293T cells with an shRNA-expressing plasmid, an envelope plasmid (pMD.G), and a packaging plasmid (pCMV-dR8.91) using calcium phosphate (Invitrogen, Carlsbad, CA, USA). The 293T cells were incubated for 18 h, followed by the replacement of the culture medium. The viral supernatants were harvested and titered at 48 and 72 h post-transfection. The cell monolayers were infected with the indicated lentivirus in the presence of polybrene and were further selected using puromycin.

### 2.5. Cell Migration and Invasion Assay

In vitro migration and invasion were investigated using a Transwell assay (Millipore, Bedford, MA, USA). For the invasion assay, transwell was additionally pre-coated with 35 µL of 3× diluted matrix matrigel (BD Biosciences Pharmingen, San Diego, CA, USA) for 30 min. A total of 2 × 10^5^ cells maintained in serum-free culture medium were added to the upper chamber of the device, and the lower chamber was filled with culture medium containing 10% FBS. After the indicated incubation periods, the cells remaining on the upper surface of the filter membrane were carefully removed using a cotton swab. The membrane was then fixed, stained with a 10× dilution of Giemsa solution (Merck, Darmstadt, Germany) and photographed. Cell motility was quantified by manually counting the cells in three random fields per filter membrane.

### 2.6. Wound Healing Assay

The wound healing assay was assessed using culture inserts (Ibidi, Martinsried, Germany). The culture inserts were transferred to plates. The cells were seeded at a density of 2 × 10^5^ cells/well and were allowed to attach. After incubation, the culture inserts were removed using sterile tweezers and washed with PBS. The plates were filled with culture medium supplemented with 2% serum to induce cell migration. The cells were photographed for quantification of closure of the exposed area. The denuded area closure was calculated by (Denuded distance _0h_ − Denuded distance _Endpoint_)/Denuded distance _0h_.

### 2.7. Western Blot Analysis

Cells were lysed using RIPA buffer containing 50 mM Tris-HCl (pH 7.4), 150 mM NaCl, 1% Triton X-100, 0.25% sodium deoxycholate, 5 mM EDTA (pH 8.0), and 1 mM EGTA supplemented with protease and phosphatase inhibitors. After 20 min of lysis on ice, cell debris was removed via microcentrifugation and the supernatants were rapidly frozen. The protein concentration was measured via the Bradford method. In my experiments, equivalent samples containing 25–100 µg of protein were loaded onto an SDS-polyacrylamide gel, separated by electrophoresis, and electrophoretically transferred from the gel onto a polyvinylidene fluoride (PVDF) membrane (Millipore, Bedford, MA, USA). After blocking with 5% nonfat milk, the membrane was hybridized with specific primary antibodies overnight at 4 °C and subsequently incubated with a corresponding horseradish peroxidase-conjugated secondary antibody for 1 h. The relative levels of proteins on the membranes were determined using an ECL-Plus Detection Kit (PerkinElmer Life Sciences, Boston, MA, USA).

### 2.8. Statistical Analysis

Estimated survival rates were determined using the Kaplan–Meier method and were compared using the log-rank test. Student’s *t* test was performed for other statistical analyses. All data are shown as the mean ± S.D. values. The *p* values within the following ranges were considered significant: * *p* < 0.05, ** *p* < 0.01, and *** *p* < 0.001. The experiments were performed at least three times and shown by the representative.

## 3. Results

### 3.1. DDX3X Expression Is Significantly Correlated with Poor Outcomes in Liver Cancer, and the Significantly Differential Gene Signature Was Identified after Comparison between Patient Groups with High and Low DDX3X Levels

**Figure 1:**
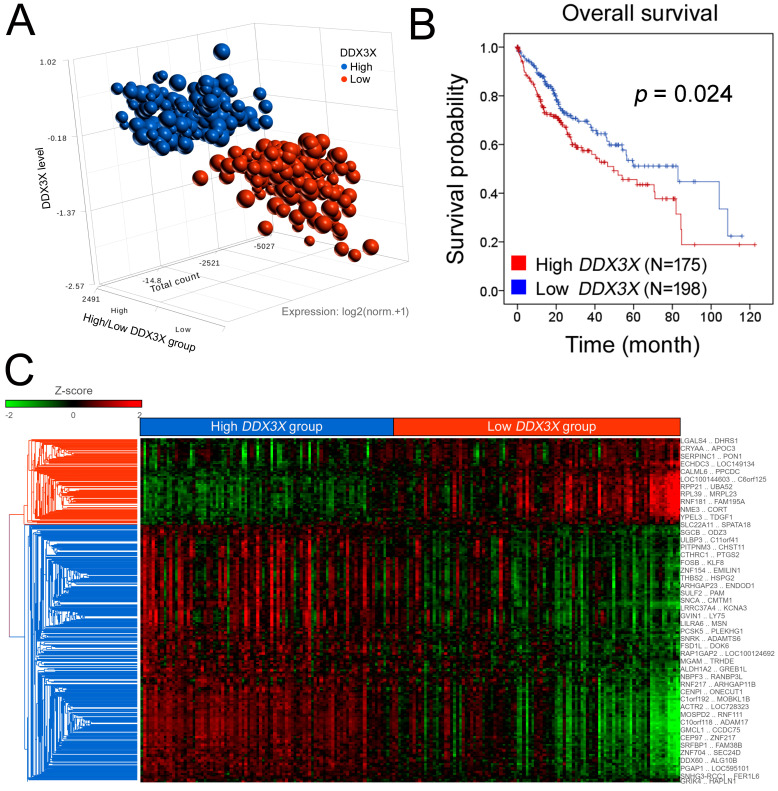

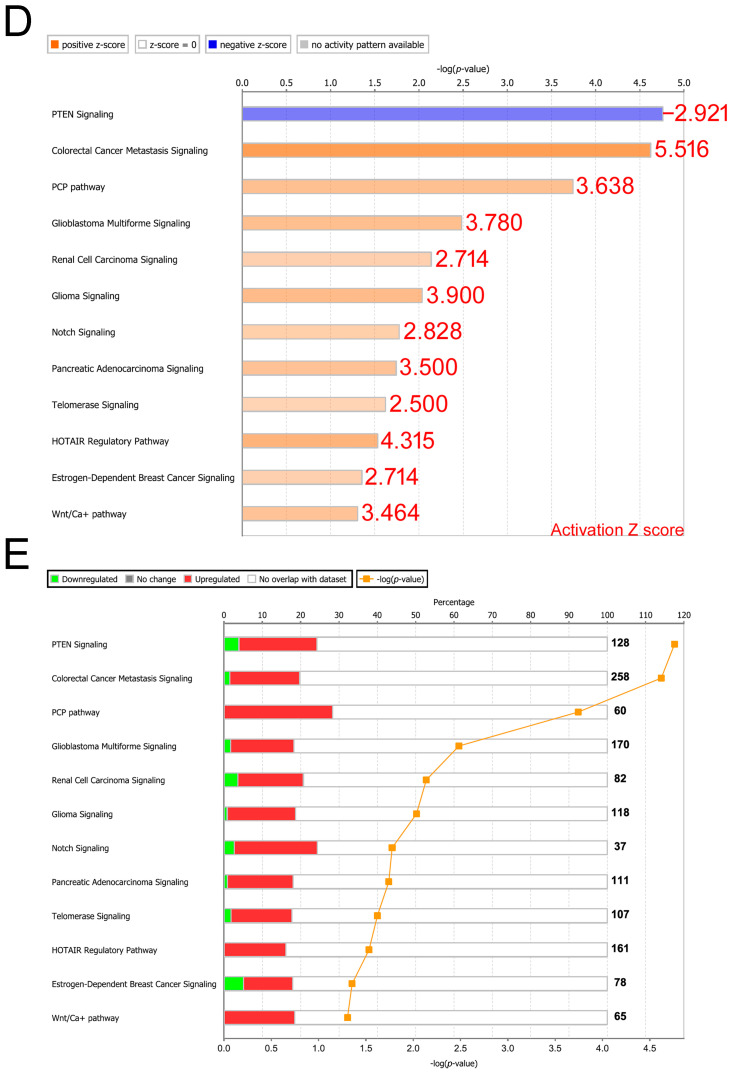
High *DDX3X* expression is associated with poor outcomes in the liver cancer cohort, and canonical pathway analysis of the *DDX3X*-dependent gene signature reveals a correlation with cancer metastasis, PCP and PTEN signaling. (**A**) A scatter plot of the indicated expression patterns in 373 liver cancer patients is shown. A total of 423 samples, including normal and tumor tissues, were retrieved from the TCGA database, and 50 adjacent normal samples were excluded prior to the analysis. The tumor samples were divided into the *DDX3X*-high and *DDX3X*-low groups based on the cut-off value around the median *DDX3X* gene expression (FPKM) in cohort. RNA-Seq data were retrieved from TCGA (Dataset ID: TCGA.LIHC.sampleMap/HiSeqV2_PANCAN, version: 2017-10-13). The RNA-Seq read count was normalized and log2 transformed. (**B**) Kaplan–Meier analysis revealed the overall survival of liver cancer patients in the *DDX3X*-high and *DDX3X*-low groups. (**C**) A total of 2890 differentially expressed gene targets in the *DDX3X*-high group were selected and are shown after hierarchical clustering (1.5-fold change, *p* value < 0.05). (**D**) Significant signaling pathways were ranked according to the −log *p* value. Activation and inactivation of the indicated signaling pathways were determined by the transformed Z score. (**E**) The percentage of genes overlapping in the indicated signaling pathways is shown. The numbers of upregulated and downregulated genes are displayed with red and green bars, respectively. The *p* values are shown on the orange line.

I first considered *DDX3X*’s clinical significance in cancer patient cohorts. A comprehensive pan-cancer study integrating cancer patients’ clinical data with RNA expression profile has been completed and released from the database: Human Protein Atlas (HPA) [14,15,16,17,18] and Kaplan–Meier plotter [19]. The prognostic data of *DDX3X* in different cancer types is listed and shown in Table 1. *DDX3X* expression predicted better survival rate in colorectal cancer, urothelial cancer, lung cancer and gastric cancer. However, *DDX3X* appeared to be an unfavorable prognostic biomarker in liver, pancreatic, breast and ovarian cancer. Among those cancer types, the up-to-date version of transcriptomic and clinical data of 373 liver cancer patients were analyzed. A scatter plot of the indicated expression patterns in the 373 patients is shown in Figure 1A.

The patients were divided into two groups with high or low *DDX3X* expression. The patients in the *DDX3X*-high group had a poor overall survival rate (*p* = 0.024, Figure 1B). Data from Cox regression analyses also indicated a trend toward *DDX3X* being useful as a prognostic factor (Table 2). These results suggest *DDX3X*’s prognostic value and indicate that further investigation regarding the molecular mechanisms altered by its overexpression is warranted.

In addition, a total of 2890 differentially expressed gene targets in the *DDX3X*-high group were selected and shown after hierarchical clustering analysis (Figure 1C and Supplementary Data Table S1).

### 3.2. Knowledge-Based Analysis of Gene Signatures Reveals the Potential for Triggering Cancer Metastasis and Progression

The gene signature identified in the *DDX3X*-high group was analyzed with knowledge-based IPA. The results obtained with the canonical pathway module of IPA identified the significant pathways ranked by overlap (log *p* value), and the activation status of each signaling was determined by the transformed Z score (Activation Z score, Figure 1D). The similarity of the high *DDX3X* gene signature to those related to the activation of colorectal cancer metastasis and planar cell polarity (PCP) signaling was identified (Figure 1D,E). The inhibition of PTEN signaling was observed (Z score: −2.921), suggesting the activation of Akt (Figure 1D).

**Figure 2:**
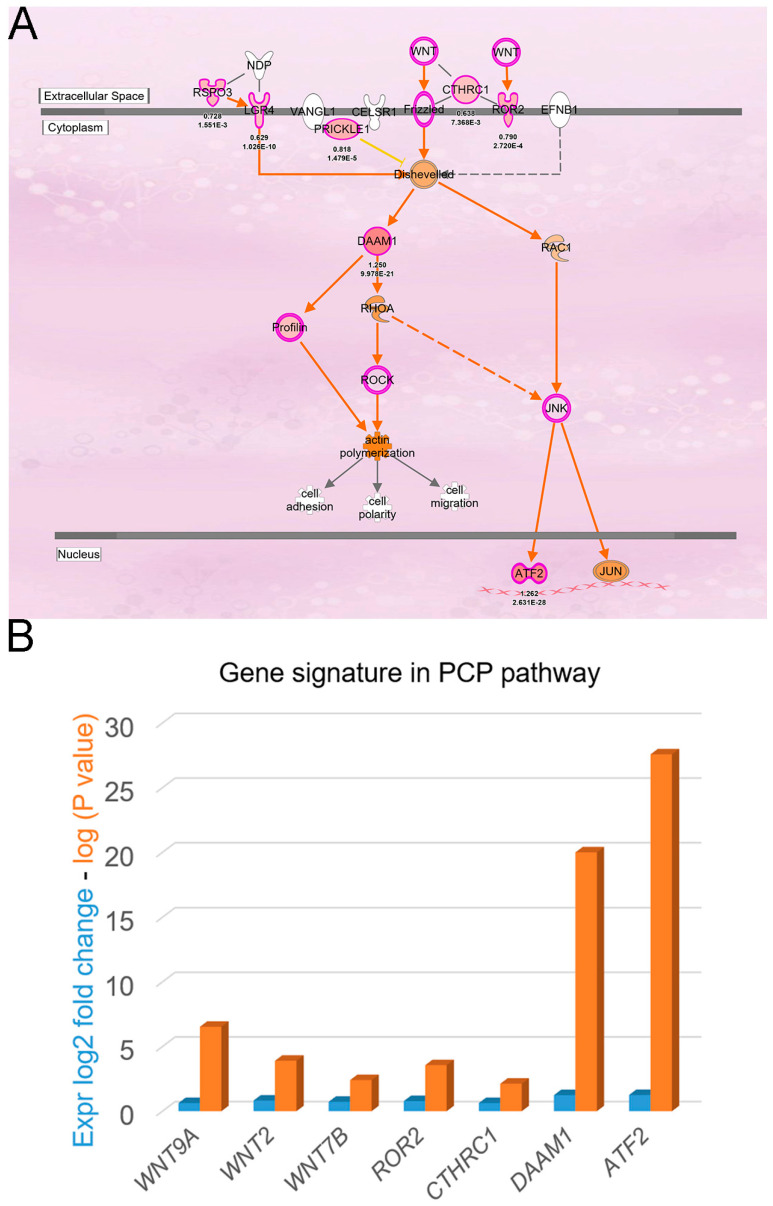
The activated PCP signaling pathway. (**A**) The signaling transduction of the PCP pathway is identified and represented after the analysis via IPA. Molecules of differential upregulation in the high DDX3X group were labeled in red. (**B**) Relative log2-transformed expressions of the principal molecules in PCP pathway are shown. The analysis was performed via Ingenuity^®^ Pathway Analysis (IPA; QIAGEN, Hilden, Germany; https://digitalinsights.qiagen.com/products-overview/discovery-insights-portfolio/analysisand-visualization/qiagen-ipa/ (accessed on 1 November 2022)).

**Figure 3:**
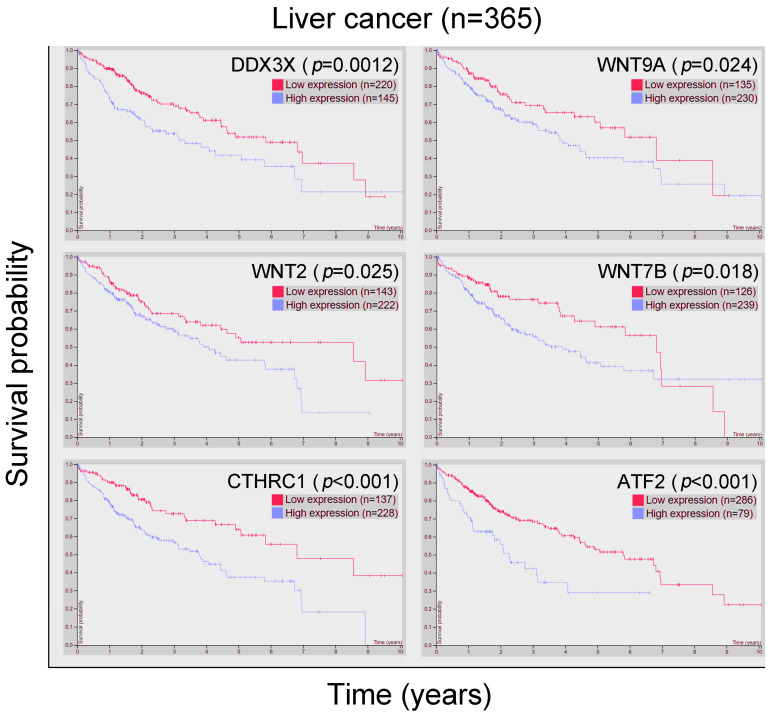
The associations of the PCP pathway-related gene signature in high *DDX3X* group with poor overall survival were analyzed. The correlation of indicated RNA levels with overall survival in liver cancer cohorts were studied and shown. Data were retrieved and analyzed from the HPA database (https://www.proteinatlas.org/ (accessed on 1 November 2022)).

**Figure 4:**
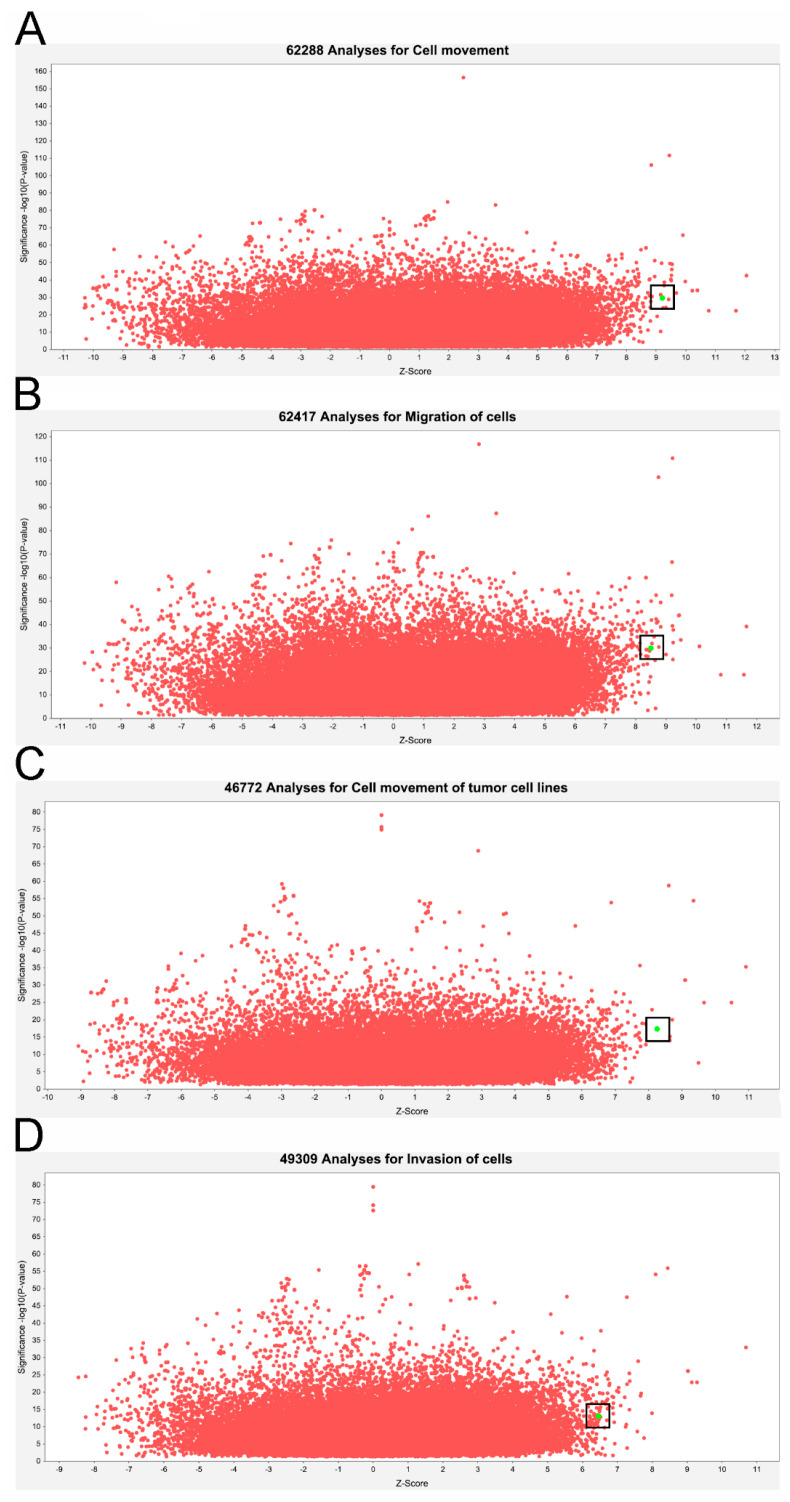
Analysis match module of IPA reveals the potential function of ATP-dependent RNA helicase DDX3X in triggering cancer cell migration. The gene signature obtained from the high *DDX3X* group was explored with the analysis match module of IPA. The gene signature was, respectively, compared with the datasets in categories of cell movement (**A**), migration of cells (**B**), cell movement of tumor cell lines (**C**) and invasion of cells (**D**). The statistical significance and activation status of the gene signature in high *DDX3X* groups were shown by green dot.

In addition, PCP pathway activation can lead to increased cancer proliferation and metastasis [20,21]. In Figure 2A, classic PCP signaling pathway was illustrated after IPA analysis of the gene signature observed in liver cancer patients with high *DDX3X* levels. Statistically highly expressed molecules including *WNT9A*, *WNT2*, *WNT7B*, *ROR2*, *CTHRC1*, *DAMM1* and *ATF2* were shown (Figure 2B). In a clinical setting, most of the aforementioned molecules were associated with poor overall survival in the liver cancer cohort (Figure 3). Moreover, the gene signature in the high *DDX3X* group was investigated by the analysis match module of IPA, which combined the curated and publicly available datasets. A significant similarity with those datasets annotated with the increased cell movement, migration of cells, cell movement of tumor cell lines and invasion of cells was detected (Table 3 and Figure 4). Hence, the transcriptomic data indicate the possibility that high *DDX3X* expression in liver cancer patients might trigger cancer progression, especially via metastasis, consistent with the observation of poor clinical outcomes (Figure 1B).

### 3.3. Knockdown of DDX3X Expression Inhibits Cell Migration and Invasion in a Liver Cancer Cell Line

**Figure 5:**
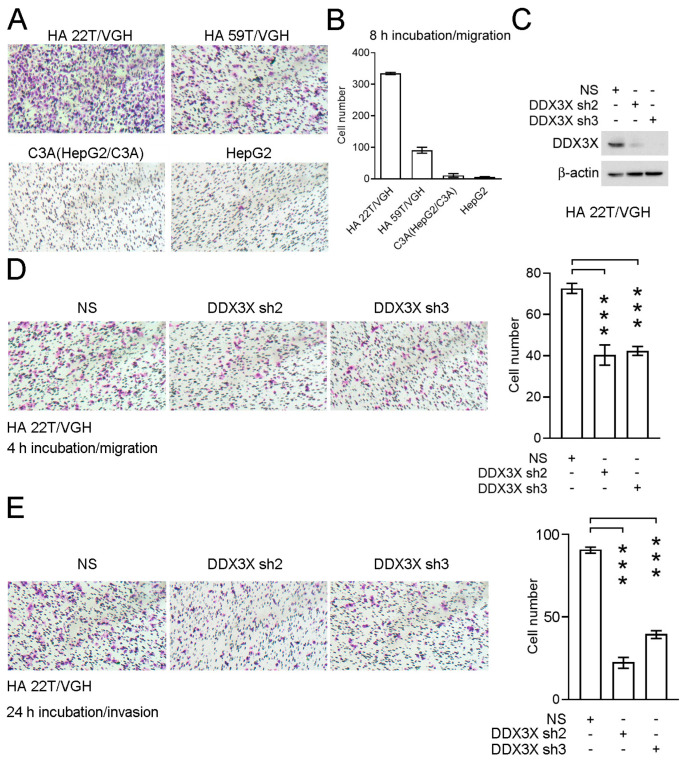
Knockdown of *DDX3X* expression inhibits HCC cell migration. (**A**) The migration ability of the indicated liver cancer cell lines was evaluated using a Transwell assay. Cells were incubated for 8 h for the migration assay in Transwell devices, followed by fixation and counting. (**B**) The numbers of migrated cells are shown. (**C**) Stable knockdown of *DDX3X* was performed in HA 22T/VGH cells. Relative ATP-dependent RNA helicase DDX3X levels were evaluated by Western blotting. NS: non-silencing control (scrambled RNA). (**D**,**E**) The migration and invasion ability of the indicated HA 22T/VGH cells were investigated in Transwell devices. *** *p* < 0.001.

Therefore, I focused on ATP-dependent RNA helicase DDX3X’s biological effect on modulating cancer cell migration. The relative cell migration levels were determined in an HCC cell line panel including HA 22T/VGH, HA 59T/VGH, C3A (HepG2/C3A) and HepG2 cells (Figure 5A). HA 22T/VGH exhibited significant malignant metastatic behavior (Figure 5B). ATP-dependent RNA helicase DDX3X expression was further stably silenced via the lentiviral transduction of two specific shRNAs in HA 22T/VGH cells (Figure 5C). Decreases in both cell migration and invasion were found after *DDX3X* silencing (*p* < 0.001, Figure 5D,E). Hence, roles of ATP-dependent RNA helicase DDX3X in regulating cancer cell migration and invasion are proposed.

### 3.4. Chaetocin Reverses the High DDX3X Expression-Mediated Gene Signature and Suppresses Liver Cancer Cell Migration

**Figure 6:**
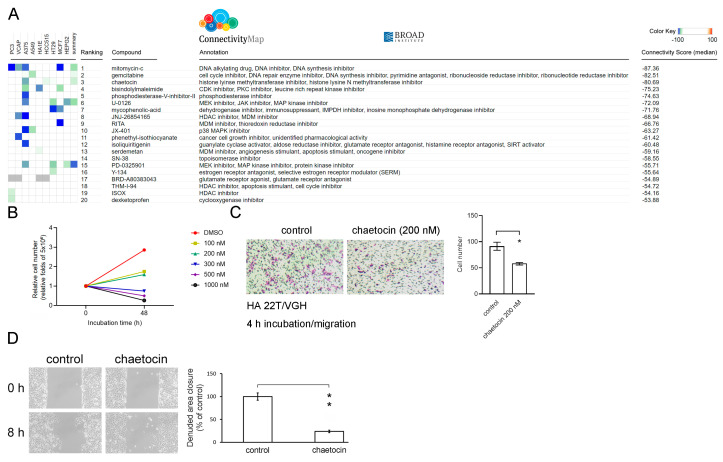
Via the L1000 platform of Connectivity Map, chaetocin is identified to reverse the high *DDX3X* expression-mediated gene signature, and chaetocin treatment suppresses HCC cell migration. (**A**) The gene signature identified in the *DDX3X*-high group versus the *DDX3X*-low group was analyzed via the L1000 platform of the Connectivity Map. The candidate drugs were ranked according to the connectivity score and are shown. (**B**) HA 22T/VGH cell numbers were determined using a trypan blue exclusion assay after 48 h of chaetocin pretreatment at the indicated concentrations. (**C**) HA 22T/VGH cell migration was evaluated using a Transwell assay of 4 h incubation and (**D**) with a wound-healing assay of 8 h incubation after 48 h of 200 nM chaetocin pretreatment. * *p* < 0.05, ** *p* < 0.01.

I further performed in silico data analysis to identify suitable compounds for inhibiting HCC progression. The updates of the Connectivity Map containing over 1.3 million L1000 profiles have been released [13]. I compared the *DDX3X* perturbation analysis with those data in the L1000 platform of the Connectivity Map to screen potential therapeutic compounds that could reverse the gene expression signature detected in the HCC patient group with high *DDX3X* expression. Candidate compounds were listed according to the negative connectivity scores determined after perturbation analysis (Figure 6A). Mitomycin-c, gemcitabine and chaetocin were the top three candidates that appeared to possess the ability to reverse the gene signature observed in the *DDX3X*-high group. Both mitomycin-c and gemcitabine have been proven to reduce cancer cell metastasis [22,23]. Chaetocin is a fungal metabolite isolated from *Chaetomium* species fungi and shows various pharmacological and biological functions, including the ability to inhibit histone lysine methyltransferase activity [24]. Chaetocin was found to repress the self-renewal of bladder cancer stem cells [25] and to act as a sensitizer of apoptosis in glioblastoma [26]. However, the biological impact of chaetocin on cancer metastasis remains unknown. Chaetocin was first selected for the next tests of therapeutic capabilities to suppress HCC cell migration in this study. As shown in Figure 6B, chaetocin treatment resulted in a dose-dependent reduction in HA 22T/VGH cell proliferation. In addition, a sublethal concentration of 200 nM chaetocin was used for the treatment of HA 22T/VGH cells. The experimental results further showed chaetocin’s inhibitory effect on cancer cell migration (Figure 6C,D).

## 4. Conclusions

**Figure 7:**
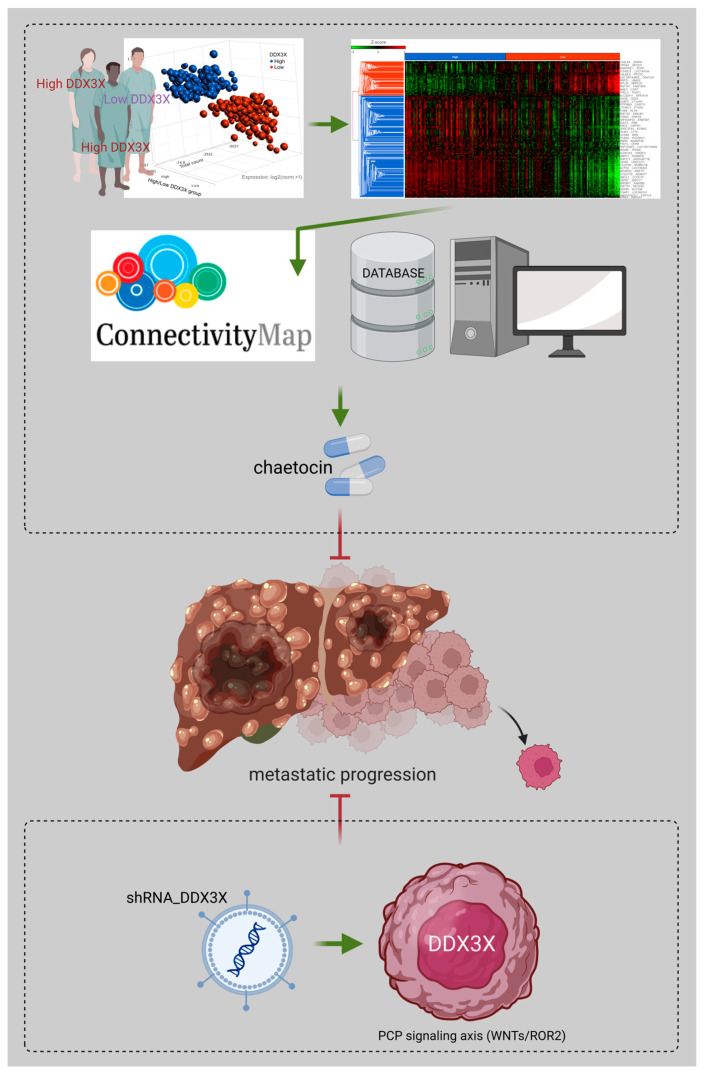
The illustration of targeting ATP-dependent RNA helicase DDX3X and reversing its downstream gene signatures-based chaetocin treatment in attenuating HCC metastatic progression.

In this study, *DDX3X* was characterized as a poor prognostic biomarker for an HCC patient cohort, and correlations of high *DDX3X* level with cancer metastasis and the activation of PCP signaling in HCC cells were discovered. *DDX3X* knockdown in the HCC cell line results in the repression of cells’ migration and invasion ability. Furthermore, my study demonstrates for the first time a high-throughput drug screening-based characterization of chaetocin and reveals its potential antimetastatic effect, especially in HCC cells with high *DDX3X* expression levels, as illustrated in Figure 7.

## 5. Discussion

In this study, I characterized *DDX3X*’s clinical significance in predicting poor hepatoma patient survival based on RNA expression data. The experimental findings indicated that ATP-dependent RNA helicase DDX3X might potentially possess functions in regulating liver cancer progression. Hence, the next urgent need is to investigate the clinical significance including the associations with patient survival rate, TNM status and clinico-pathological features at a protein level in a patient cohort of HCC.

In this research study, I first observed the potentially therapeutic application of chaetocin for hepatocellular carcinoma in terms of precision medicine; that is, especially for those cancer patients displaying high *DDX3X* expression levels. Actually, ATP-dependent RNA helicase DDX3X appeared to be modulated by several compounds and cytokines via direct and/or indirect interactions. In liver hepatocellular cells, previous findings indicated that 5-HT treatment could augment 5-HT receptor 7-mediated *DDX3X* promoter activity as well as the induction of an innate immunity to abolish hepatitis B virus (HBV) infection [27]. Another research finding in hepatocellular HepG2 cells demonstrated that the addition of tazemetostat, SP2509, decitabine and trichostatin A led to the downregulation of *DDX3X* RNA [28]. Inhibiting ATP-dependent RNA helicase DDX3X’s ATP binding domain with the small molecule RK-33 was one of choices, and the effect was discovered to synergize with radiotherapy to reduce tumor cells’ proliferation in vitro and in vivo [29]. In a cancer-related study, RK-33 treatment was indicated to inactivate the WNT signaling axis and induce cell cycle G1 phase arrest, leading to cell apoptosis in lung cancer [30]. In addition, 1,3,4-thiadiazole is another ATPase activity inhibitor for ATP-dependent RNA helicase DDX3X, synthesized for blocking HIV-1-mediated effects [31]. The design of ATP-competitive inhibitors reported that FE15 and FE109 appeared to block helicase and the ATPase function of ATP-dependent RNA helicase DDX3X, along with having the capability to reduce the HIV viral load in peripheral blood mononuclear cells [32]. Furthermore, doxorubicin is considered to be one of the ATP-dependent RNA helicase DDX3X inhibitors via an in silico molecular docking approach which showed a potential interaction with common amino acid residues Tyr200/Thr201 and unique amino acid residue Thr198 via doxorubicin. The realistic function was also corroborated in oral squamous cell carcinoma H357 cells. *DDX3X* downregulation and the decreases in ATP hydrolysis, inorganic phosphate release and cancer proliferation were reported [33]. High throughput virtual screening was performed to identify ATP-dependent RNA helicase DDX3X inhibitors, in which Ketorolac salt had been characterized as a bioactive compound for its ability to bind with ATP-dependent RNA helicase DDX3X. The interaction further appeared to suppress the tumor growth in oral cancer [34]. In contrast to the aforementioned strategies, I first explored the drug candidates, focusing on the basis of comprehensively reversing *DDX3X*-dependent downstream gene signatures, and characterized chaetocin as one of potential targets for therapeutics in liver cancer. Nevertheless, several molecules related to upstream regulation and the direct interaction of ATP-dependent RNA helicase DDX3X might be noticed. AGR2 is a member of the protein disulfide isomerase family, which acts as a proto-oncogene. AGR2 was found to trigger cancer metastasis in animal models, and the interaction of ATP-dependent RNA helicase DDX3X with AGR2 has been characterized at protein level [35]. In addition, fibronectin 1 was uncovered to upregulate the expression of human *DDX3X* at an RNA level in cultured HUVEC cells in a microarray analysis [36]. Ginsenoside Rg3 stimulus-enhanced *DDX3X* upregulation and the Akt-p53-dependent *DDX3X* promoter transactivation were detected, along with the activation of innate immune response via the TBK1-IKKε-IRF3 pathway [37].

Chaetocin was identified based on the comparison of gene signatures derived from those cancer patients displaying high *DDX3X* levels with the datasets in the ConnectivityMap database (negative connectivity score, Figure 6A). The same gene signature was simultaneously analyzed via IPA. A significant signaling axis, the PCP pathway, was characterized according to the activation Z score (Figure 1D), which is a pivotal stimulus in triggering cancer progression [20,21]. Therefore, the PCP signaling axis might serve as a potential route inhibited by chaetocin to reverse *DDX3X*-mediated effects because of the overlap of similar downstream molecules.

## Figures and Tables

**Table 1:** Prognostic significance of *DDX3X* in pan-cancer.

Symbol	Cancer Type	Prognosis	Endpoint	*p* Value	Case	Dataset	Method	Probe ID
*DDX3X*	Glioma	-	Overall survival	N.S.	153	TCGA	RNA Seq	
*DDX3X*	Thyroid Cancer	-	Overall survival	N.S.	501	TCGA	RNA Seq	
*DDX3X*	Lung Cancer	-	Overall survival	N.S.	994	TCGA	RNA Seq	
*DDX3X*	Colorectal Cancer	Good	Overall survival	0.008	597	TCGA	RNA Seq	
*DDX3X*	Head and Neck Cancer	-	Overall survival	N.S.	499	TCGA	RNA Seq	
*DDX3X*	Stomach Cancer	-	Overall survival	N.S.	354	TCGA	RNA Seq	
*DDX3X*	Liver Cancer	Poor	Overall survival	0.0012	365	TCGA	RNA Seq	
*DDX3X*	Pancreatic Cancer	Poor	Overall survival	0.033	176	TCGA	RNA Seq	
*DDX3X*	Renal Cancer	-	Overall survival	N.S.	877	TCGA	RNA Seq	
*DDX3X*	Urothelial Cancer	Good	Overall survival	0.029	406	TCGA	RNA Seq	
*DDX3X*	Prostate Cancer	-	Overall survival	N.S.	494	TCGA	RNA Seq	
*DDX3X*	Testis Cancer	-	Overall survival	N.S.	134	TCGA	RNA Seq	
*DDX3X*	Breast cancer	Poor	Overall survival	0.024	1075	TCGA	RNA Seq	
*DDX3X*	Cervical Cancer	-	Overall survival	N.S.	291	TCGA	RNA Seq	
*DDX3X*	Endometrial Cancer	-	Overall survival	N.S.	541	TCGA	RNA Seq	
*DDX3X*	Ovarian Cancer	-	Overall survival	N.S.	373	TCGA	RNA Seq	
*DDX3X*	Melanoma	-	Overall survival	N.S.	102	TCGA	RNA Seq	
*DDX3X*	Breast cancer	Poor	Relapse-free survival	<0.001	4929	E-MTAB-365, E-TABM-43, GSE: 11121, 12093,	Array	201210_at
						12276, 1456, 16391, 16446, 16716, 17705, 17907,		
						18728, 19615, 20194, 20271, 2034, 20685, 20711,		
						21653, 22093, 25066, 2603, 26971, 29044, 2990,		
						31448, 31519, 32646, 3494, 36771, 37946, 41998,		
						42568, 43358, 43365, 45255, 4611, 46184, 48390,		
						50948, 5327, 58812, 61304, 65194, 6532, 69031,		
						7390, 76275, 78958, 9195		
*DDX3X*	Ovarian cancer	Poor	Progression-free survival	<0.001	1435	GSE: 14764, 15622, 18520, 19829, 23554, 26193,	Array	201210_at
						26712, 27651, 30161, 3149, 51373, 63885, 65986,	RNA Seq	
						9891, TCGA (N = 565)		
*DDX3X*	Lung cancer	Good	Overall survival	<0.001	1925	CAARRAY, GSE: 14814, 19188, 29013, 30219,	Array	201210_at
						31210, 3141, 31908, 37745, 43580, 4573, 50081,	RNA Seq	
						8894, TCGA (N = 133)		
*DDX3X*	Gastric cancer	Good	Post progression survival	<0.001	498	GSE: 14210, 15459, 22377, 29272, 51105, 62254	Array	201210_at

Survival data were collected from databases The Human Protein Atlas, TCGA and Kaplan–Meier plotter. N.S.: no significance.

**Table 2:** Cox regression analysis of correlations of pathological stage, TNM prognostic factors and *DDX3X* expression with overall survival in 373 liver cancer patients.

Variable	Comparison	HR (95% CI)	*p* Value
Sex	M:F	0.800 (0.562–1.141)	0.218
Stage	3–4:1–2	2.485 (1.714–3.603)	<0.001
T	T3–4:T1–2	2.578 (1.812–3.667)	<0.001
N	N1:N0	2.012 (0.493–8.212)	0.330
M	M1:M0	4.055 (1.274–12.906)	0.018
*DDX3X*	High:Low	1.485 (1.050–2.099)	0.025

Abbreviations: F: female; M: male; HR: hazard ratio; CI: confidence interval. T: tumor; N: nodes; M: metastasis.

**Table 3:** Diseases and functions of dataset: *DDX3X*_high/low.

Ranking	Categories	Diseases/Functions Annotation	Predicted Activation State	Activation z-Score
1	Cellular Movement	Cell movement	Increased	9.208
2	Cellular Movement	Migration of cells	Increased	8.506
3	Cellular Assembly and Organization, Cellular Function and Maintenance	Organization of cytoplasm	Increased	8.472
4	Cellular Assembly and Organization, Cellular Function and Maintenance	Organization of cytoskeleton	Increased	8.43
5	Cellular Movement	Cell movement of tumor cell lines	Increased	8.254
6	Tissue Morphology	Quantity of cells	Increased	8.25
7	Cell Morphology, Cellular Assembly and Organization, Cellular Function and Maintenance	Formation of cellular protrusions	Increased	8.077
8	Cellular Assembly and Organization, Cellular Function and Maintenance	Microtubule dynamics	Increased	7.699
9	Cellular Development, Cellular Growth and Proliferation, Nervous System Development and Function, Tissue Development	Development of neurons	Increased	6.967
10	Cellular Movement	Homing of cells	Increased	6.705
11	Cellular Function and Maintenance	Cellular homeostasis	Increased	6.55
12	Cellular Movement	Invasion of cells	Increased	6.461
13	Cardiovascular System Development and Function	Development of vasculature	Increased	6.43
14	Cardiovascular System Development and Function, Organismal Development	Angiogenesis	Increased	6.418
15	Cellular Movement	Chemotaxis	Increased	6.411

## Data Availability

All necessary data have been provided in this manuscript and Supplementary Data.

## Supplementary Materials

The following supporting information can be downloaded at: https://www.mdpi.com/article/10.3390/cells12121628/s1, Table S1: Supplementary data_Table S1.
